# School teachers' awareness of internet addiction in elementary school students: a regional survey in Japan

**DOI:** 10.3389/fpsyt.2023.1187387

**Published:** 2023-07-13

**Authors:** Kentaro Kawabe, Fumie Horiuchi, Rie Hosokawa, Kiwamu Nakachi, Junya Soga, Shu-ichi Ueno

**Affiliations:** ^1^Department of Child Psychiatry, Ehime University Graduate School of Medicine, Toon, Japan; ^2^Department of Neuropsychiatry, Ehime University Graduate School of Medicine, Toon, Japan

**Keywords:** internet addiction, elementary school, SNS, prevention, students, gaming disorder

## Abstract

In recent years, concerns about internet addiction (IA) among children have been increasing. This study focused on the awareness of IA in elementary school teachers. A web-based anonymous survey was conducted in November 2021. The participants completed an original questionnaire about their awareness of IA. The participants were divided into three groups based on their positions in the classroom: class teachers, support teachers, and administrative teachers. Out of 283 participants, over 70% had not approached students with IA and had little practical knowledge about the disorder. Support and administrative teachers had more opportunities to interact with students with IA than class teachers (*p* < 0.001 in both cases). Support teachers had more opportunities to ask their colleagues about IA than class teachers (*p* < 0.01); similarly, administrative teachers also had more opportunities to discuss IA with colleagues than class teachers (*p* = 0.04). Preventive interventions are recommended for people who communicate with children with IA. Students with IA might cause anxiety among teachers; therefore, preventive education strategies should be implemented with the cooperation of psychiatrists, psychologists, and public health nurses.

## 1. Introduction

In Japan, almost all elementary, junior, and senior high schools have established a one-to-one computer–student ratio and developed a high-speed, large-capacity communication network. The information and communication technology (ICT) environment was accelerated as a countermeasure against the spread of COVID-19 from the end of 2020, and most of the abovementioned costs were subsidized by the Japanese government (1). The internet enables educational progress in schools, but internet addiction (IA) is a rising concern among the youth. The concept of IA is rooted in behavioral addiction and the addictive nature of various internet contents such as games, video clips, and social media (2). Excessive social media usage could be a risk factor for mental health problems, including depression, anxiety, and addiction (2). We previously reported a negative association between IA in adolescents and their sleep habits and mental states (3). According to a large-scale school-based study in an elementary school in Japan, IA and risky behaviors were common among children, and IA was associated with their own unhealthy lifestyles, including prolonged internet use, late bedtime, physical inactivity, and skipping breakfast as well as family and social environments, such as no rules at home and lack of close friends in real life and child–parent interactions (4). The negative effects of IA have been reported in children, such as elementary school students (5). Not only parents but also teachers are most likely to be an important source of social support for elementary school students (6). The quality of student–teacher relationships is a key factor that fosters or undermines students' school adjustment; for example, if the students perceive that their teachers support them and care about them, they will show better academic performance and fewer problem behaviors (7). Hence, supervision and guidance from parents and teachers are needed to help children develop good internet habits and minimize the negative effects of internet usage. However, to the best of our knowledge, no study has examined the awareness levels of teachers regarding IA and supervision, guidance, and preventive programs for students and their parents. Therefore, this study aimed to determine the awareness of IA using a survey targeting elementary school teachers.

## 2. Methods

A web-based anonymous survey was conducted in November 2021. The teachers at public elementary schools in Ehime Prefecture were included. Ehime Prefecture is located in western Japan and has a population of 1,326,000 and 8,000 in 2021. There were 290 public elementary schools in Ehime with about 66,000 students. The original questionnaire consisted of seven items, with yes/no responses:

1. Have you had the opportunity to interact with students with IA?
2. Have you ever been consulted by a parent?
3. Have you ever instructed individual students about using the internet?
4. Have you ever provided guidance to parents on their children's internet usage?
5. Do you have knowledge about IA?
6. Have you asked colleagues about IA?
7. Do you think education on preventing IA is necessary?

No consensus has been reached on the relevant criteria of addictive internet use. In this study, IA was defined as unspecified overuse of the internet using different applications (8). The survey was administered via the web, along with an explanation. The participants filled out an informed consent form online. The study was approved by the Institutional Review Board (IRB) of Ehime University Graduate School of Medicine (IRB No. 1809010). Categorical variables were expressed as numbers and percentages. The participants were divided into three groups based on the teachers' positions in the classroom: class teacher, support teacher (school nurse or teacher in special classes), and teachers in administrative positions, such as principal and vice principal. A binary logistic regression analysis was performed by dividing the teachers according to age and teaching position to determine the factors associated with each question. The *P*-values of < 0.05 (two-sided) were considered to be statistically significant. All statistical analyses were performed with SPSS Statistics 26.0 (IBM Corp., Armonk, NY, USA).

## 3. Results

**Figure 1:**
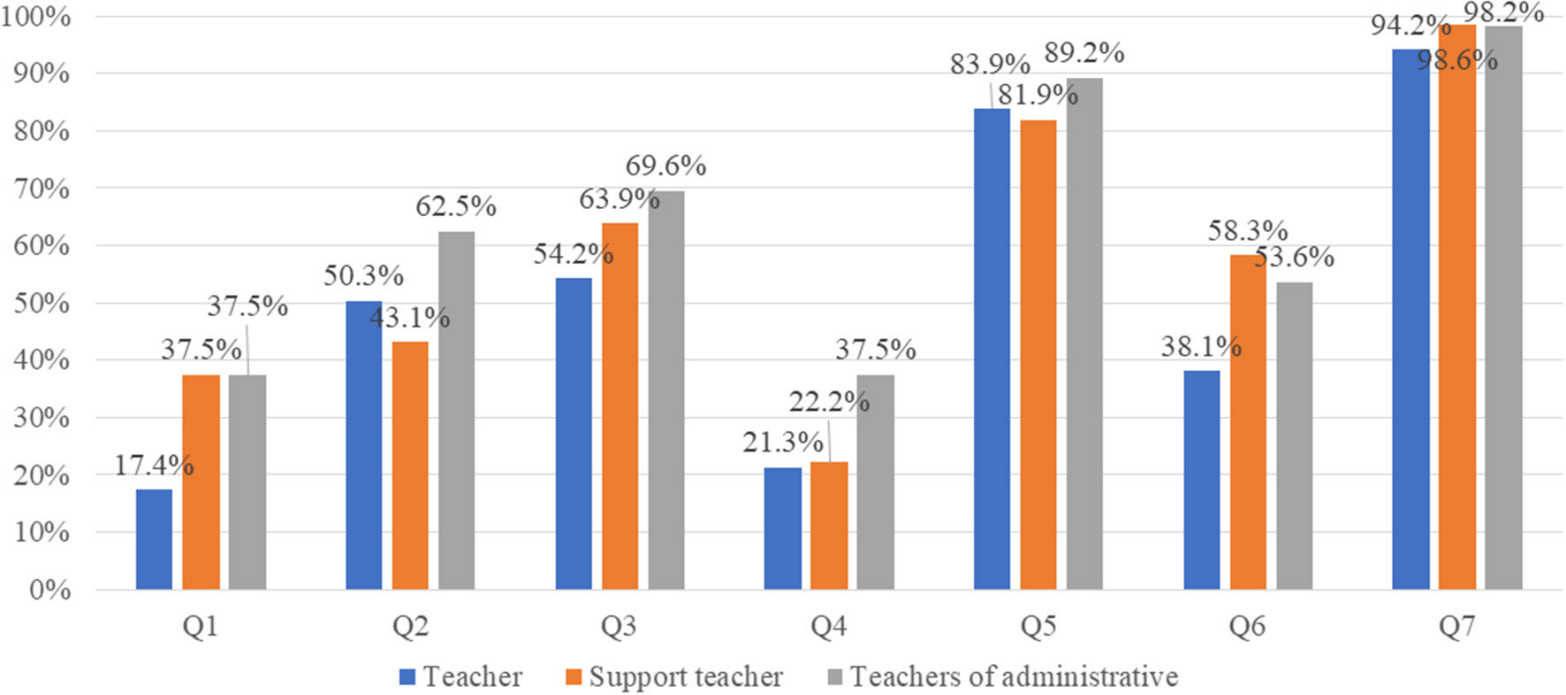
Proportions of teachers who responded yes to each item. Values are expressed as percentages.

Overall, 283 participants (120 male subjects and 163 female subjects) completed the survey. The most frequent respondents were class teachers (*n* = 155), followed by support teachers (*n* = 72) and administrative teachers (*n* = 56). Table 1 shows the characteristics of the participants in these three groups. Figure 1 shows the results of the questionnaire responses of the three groups. Regarding Q1, more than 70% of the teachers had not approached the students with IA, and only 17.4% of the teachers interacted with students who had IA. Table 2 shows the results of logistic regression analysis examining the factors associated with age group and teacher's position. Support teachers had more opportunities to interact with students with IA than class teachers [odds ratio (OR): 3.37, 95% confidence interval (CI): 1.74–6.55, *p* < 0.001]. Similarly, administrative teachers also had more opportunities to interact with students than class teachers (OR: 3.73, 95% CI: 1.79–7.78, *p* < 0.001). Teachers in their 50s had fewer opportunities to interact with students with IA than those in their 20s (OR: 0.44, 95% CI: 0.20–0.96, *p* = 0.04) (Q1). Administrative teachers had more opportunities to consult parents about their children's internet use than class teachers (OR: 2.31, 95% CI: 1.15–4.65, *p* = 0.02) (Q4). Support teachers had more opportunities to ask their colleagues about IA than class teachers (OR: 2.37, 95% CI: 1.32–4.27, *p* < 0.01); similarly, administrative teachers also had more opportunities to discuss IA with colleagues than class teachers (OR: 1.96, 95% CI: 1.03–3.75, *p* = 0.04). Teachers in their 30s had more opportunities to ask their colleagues about IA than those in their 20s (OR: 2.56, 95% CI: 1.20–5.64, *p* = 0.02) (Q6). Although there was no significant ratio difference among the three groups, more than 90% stated that education for preventing IA was needed (Q7).

**Table 1:** Characteristics of the participants.

	**Total**	**Class teacher**	**Support teacher**	**Administrative teacher**
Number, *n* (%)	283 (100)	155 (100)	72 (100)	56 (100)
Male	120 (42.4)	73 (47.1)	8 (11.1)	39 (69.6)
**Age range**				
20s	58 (20.5)	43 (27.7)	11 (15.3)	4 (7.1)
30s	55 (19.4)	37 (23.9)	11 (15.3)	7 (12.5)
40s	53 (18.7)	24 (15.5)	17 (23.6)	12 (21.4)
Above 50	117 (41.3)	51 (32.9)	33 (45.8)	33 (58.9)

Table 2Logistic regression analysis examining the factors associated with age group and teacher's position.

**Table:** 

**Yes**, ***n*** **(%)**	**Q1**		**Q2**		**Q3**		**Q4**	
	**75 (26.5)**		**144 (50.9)**		**169 (59.7)**		**70 (24.7)**	
	**OR (95%CI)**	***p***	**OR (95%CI)**	***p***	**OR (95%CI)**	***p***	**OR (95%CI)**	***p***
**Age group**								
20s	Reference		Reference		Reference		Reference	
30s	0.98 (0.42–2.27)	0.96	1.36 (0.64–2.86)	0.43	1.45 (0.69–3.07)	0.33	2.23 (0.92–5.44)	0.77
40s	0.64 (0.27–1.53)	0.32	0.91 (0.42–1.95)	0.80	2.23 (0.99–4.97)	0.05	1.63 (0.64–4.13)	0.30
Above 50	0.44 (0.20–0.96)	0.04^*^	0.86 (0.45–1.65)	0.65	1.23 (0.64–2.37)	0.54	1.17 (0.51–2.71)	0.71
**Teacher's position**								
Class teachers	Reference		Reference		Reference		Reference	
Support teachers	3.37 (1.74–6.55)	< 0.001 ^***^	0.79 (0.44–1.40)	0.41	1.42 (0.79–2.56)	0.24	1.08 (0.54–2.15)	0.84
Administrative teachers	3.73 (1.79–7.78)	< 0.001^***^	1.79 (0.93–3.43)	0.08	1.85 (0.94–3.63)	0.08	2.31 (1.15–4.65)	0.02^*^

**Table:** 

	**Q5**		**Q6**		**Q7**	
**Yes**, ***n*** **(%)**	**239 (84.5)**		**131 (46.3)**		**272 (96.1)**	
	**OR (95%CI)**	***p***	**OR (95%CI)**	***p***	**OR (95%CI)**	***p***
**Age group**						
20s	Reference		Reference		Reference	
30s	1.67 (0.63–4.43)	0.30	2.56 (1.20–5.64)	0.02^*^	2.77 (0.28–27.69)	0.39
40s	2.23 (0.77–6.51)	0.14	1.61 (0.73–3.55)	0.24	0.65 (0.12–3.49)	0.62
Above 50	1.63 (0.71–3.75)	0.25	1.22 (0.62–2.43)	0.56	1.09 (0.23–5.24)	0.91
**Teacher's position**						
Class teachers	Reference		Reference		Reference	
Support teachers	0.80 (0.37–1.70)	0.56	2.37 (1.32–4.27)	< 0.01^**^	4.84 (0.59–39.72)	0.14
Administrative teachers	1.41 (0.53–3.77)	0.49	1.96 (1.03–3.75)	0.04^*^	3.73 (0.44–31.31)	0.23

Q, question; OR, odds ratio; CI, confidence interval.

*** for *p* < 0.001,

** for *p* < 0.01, and

* for *p* < 0.05.

## 4. Discussion

This study aimed to examine teachers' attitudes toward students with IA. Our results showed that support and administrative teachers had more opportunities to interact with students and consult colleagues about IA; class teachers had fewer interactions with students with IA than support and administrative positions teachers. The age range coefficients are rarely significant and inconsistent, making the interpretation challenging.

Although the effect of IA on physiological and psychological health is tremendous, the prevalence is relatively low (4.2%) among elementary school students (4). However, a previous study reported an increase in screen time in the general population during the COVID-19 pandemic (9). In this study, only 17.4% of the class teachers had approached or interacted with students with IA. Although a simple comparison between current prevalence data and this survey may not be appropriate, the prevalence of IA among elementary school students may be higher than in the past report.

Although approximately half of the teachers were consulted by parents about IA (50.9%), many teachers were not able to offer advice to parents (24.7%) according to Q2 and Q4. IA was not categorized as a psychiatric disorder in the latest DSM or ICD; only gaming disorder is categorized as a psychiatric disorder according to the ICD-11. According to research on inclusive education, Japanese teachers have high anxiety levels. They are critical about including children with disabilities in their classrooms although many teachers believe inclusive education is necessary (10). Similarly, students with IA might also be a source of anxiety for teachers. Among typical occupations, teachers commonly report high levels of occupational stress (11). Class teachers, in particular, face multiple demands from students, parents, colleagues, and administrative teachers (12). Therefore, according to our results, support and administrative teachers can try to assist and advise class teachers, students, and parents facing problems related to internet usage.

Almost all participants stated that education for preventing IA was needed (Q7). Relatively brief school-based services seem to have positive effects on elementary-aged children; some programs go beyond the primary role of teachers (13). Hence, preventive education for IA should not be carried solely by school teachers, but the cooperation of related institutions and psychiatrists, psychologists, and public health nurses is needed. A research-based structured model using a widespread peer-support scheme, specially designed courses and seminars, testing for potential problems, and counseling services has been proposed to combat cyberbullying in Japan (14). Constructing a model that focuses not only on cyberbullying but also on IA may be beneficial. Although the literature on preventing IA is scarce, preventive interventions are recommended for people who communicate with children suffering from IA, such as parents, teachers, peers, and others close to them (15). However, there is insufficient information on the guidelines or general prevention methods in Japan. A recent study reported that a prevention program involving educational workshops would positively impact and help reduce children's susceptibility to IA (16). Specifically, peer-to-peer approaches, media literacy enhancement, reduction of co-morbid symptoms and negative psychosocial consequences, awareness-raising, and emphasis on positive psychology are effective prevention programs in school (17). In particular, school-based prevention programs for intermediate elementary students can help improve intermediate digital health literacy (18). Prevention programs in collaboration with school teachers should be implemented in future studies.

A methodological limitation of the study is that it was web-based; thus, only users with internet access could complete it. In addition, the survey was anonymous, where individual and school names were not revealed, so an accurate response rate could not be calculated. The relatively low participant number makes the results less generalizable and may not represent all teachers' opinions. Another limitation is that this study used a non-validated original questionnaire that limited the interpretability of the results and, therefore, the estimates and had a risk of bias.

## Data availability statement

The original contributions presented in the study are included in the article/supplementary material, further inquiries can be directed to the corresponding author.

## Ethics statement

The study was approved by the Institutional Review Board of Ehime University Graduate School of Medicine (IRB No. 1809010). Participants filled out the informed consent form online.

## Author contributions

KK designed the study, performed the statistical analyses, analyzed and interpreted the data, drafted the manuscript, and prepared the first draft of the article. S-iU supervised this study. All authors contributed substantially to the analysis and interpretation of clinical data and contributed to and approved the final version of the manuscript.
